# Chikungunya-Driven Gene Expression Linked to Osteoclast Survival and Chronic Arthralgia

**DOI:** 10.3390/idr16050073

**Published:** 2024-09-20

**Authors:** Alysson Henrique Urbanski, Vanessa E. Maso, Felipe M. Martins, André Guilherme da Costa-Martins, Ana Paula B. do Nascimento Oliveira, Helder I. Nakaya

**Affiliations:** 1 School of Pharmaceutical Sciences, University of São Paulo, São Paulo 05508-020, Brazilvanessa.escolano.maso@gmail.com (V.E.M.); felipemartins1305@usp.br (F.M.M.); agcostamartins@gmail.com (A.G.d.C.-M.); anapaula.oliveira@cchmc.org (A.P.B.d.N.O.); 2 Micromanufacturing Laboratory, Institute for Technological Research—IPT, São Paulo 05508-901, Brazil; 3 Hospital Israelita Albert Einstein, São Paulo 05653-000, Brazil

**Keywords:** chikungunya fever, rheumatoid arthritis, RANK signaling, CDR3, B cells, T cells

## Abstract

Chikungunya fever (CHIKF), caused by the Chikungunya virus (CHIKV), manifests as acute febrile illness often associated with polyarthritis and polyarthralgia. Although the acute symptoms resolve within two weeks, many patients experience prolonged joint pain and inflammation, resembling rheumatoid arthritis (RA). This study aimed to identify molecular markers related to joint pain and chronicity in CHIKV-infected individuals by analyzing blood transcriptomes using bulk RNA sequencing. B- and T-cell receptor (BCR and TCR) diversity was assessed through computational analysis of RNA-seq data, revealing a significant reduction in CDR3 diversity in CHIKV-infected individuals compared to healthy controls. This reduced diversity was associated with the upregulation of genes involved in osteoclast differentiation and activation, particularly through the RANK/RANKL signaling pathway. These findings suggest a potential link between immune dysregulation and enhanced osteoclast activity, which may contribute to the persistence of joint pain in chronic CHIKF. Targeting osteoclast-related pathways could offer therapeutic strategies for managing chronic symptoms in CHIKF patients.

## 1. Introduction

The Chikungunya virus (CHIKV), transmitted to humans by *Aedes* mosquitoes, causes a febrile illness known as Chikungunya fever (CHIKF). Originally from East Africa, CHIKF is now present in more than 60 countries in four continents. The infection commonly resolves in two weeks. The acute phase of CHIKF typically lasts around one week and is characterized by the sudden onset of fever, polyarthralgia, skin rash, and myalgia [1]. Mortality rates are no higher than 0.8%, but CHIKF can progress to a chronic state characterized by persistent joint pain and inflammation that can last for months, leading to mobility problems and decreased quality of life [2]. A recent study found that one out of eight patients developed long-term joint pain, up to 40 months [3]. These chronic symptoms are associated with large economic impacts, especially in countries in the global south countries [1].

CHIKF has been previously linked to rheumatoid arthritis (RA) due to similar inflammatory processes. CHIKV infection of fibroblast-like synoviocytes induces the expression of chemotactic agents, including RANKL, IL-6, IL-8, and MCP-1, which recruit and differentiate phagocytes into osteoclast-like cells. These cells produce TNF-α and IL-6, the primary mediators of arthritis [4]. Osteoclasts play a crucial role in bone erosion in RA, and there is evidence of increased osteoclast precursor abundance and activity in peripheral blood cells of RA patients [5]. The balance between osteoclast and osteoblast activity determines the degree of bone erosion in RA [5]. CHIKV infection of osteoblasts increases RANKL and IL-6 production while decreasing osteoprotegerin (OPG) production, promoting osteoclastogenesis [6].

Given the immune response links between CHIKF and RA, this study aimed to analyze publicly available RNAseq data in order to identify immune-related blood markers in CHIKV-infected individuals that are shared with chronic RA patients and could potentially explain the underlying causes of joint pain and chronicity in CHIKF. The discovery of such common biological processes could open the possibility for new and improved treatments for chronic joint pain in CHIKF patients.

## 2. Materials and Methods

### 2.1. RNA-Seq Data

Two publicly available RNA-Seq datasets were utilized for the analyses. The Chikungunya dataset, as detailed in reference [7], includes 24 CHIKV-infected patients, all in the acute phase of the disease, with symptom onset occurring 0 to 4 days before sample collection, and 13 non-infected patients. Real-time reverse transcription polymerase chain reaction (RT-PCR) was performed to test for CHIKV. Peripheral blood RNA extraction and sequencing using HiSeq Illumina 1500 are described in the original article [7].

The Dengue dataset, described in reference [8] and used as a comparative control, consists of 18 DENV-infected individuals and 16 non-infected individuals. Acute Dengue was confirmed using IgG ELISA, virus isolation, and RT-PCR. Peripheral blood RNA extraction and sequencing using Illumina NovaSeq 6000 are detailed in the original article [8].

**Figure 1:**
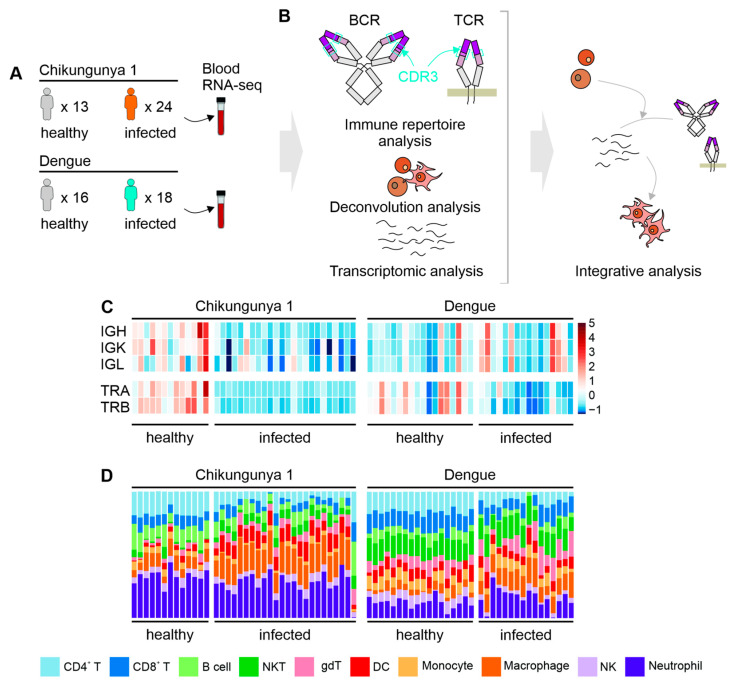
Study design, CDR3 diversity, and cell abundance. (**A**) Number of samples and group division in each dataset. (**B**) Workflow for integrating immune repertoire, deconvolution, and transcriptomic analysis from RNA-seq. (**C**) CDR3 Chao1 diversity for BCRs (IGH, IGK, and IGL) and TCRs (TRA and TRB), normalized by z-score for each chain within the same dataset. (**D**) Summary of the estimated cells in each sample.

Comprehensive sample quality control was conducted on all samples. During the estimations of BCR and TCR CDR3 diversity, clear outliers were identified and excluded to prevent any bias in the analysis. These outliers displayed significantly different immune repertoire diversity patterns that could not be attributed to any other variables reported in the original studies, such as sequencing metrics or clinical symptoms. Both datasets encompass individuals within specific age ranges: 18 to 70 years for the Chikungunya dataset and 15 to 72 years for the Dengue dataset. A summary of the datasets and samples is presented in Figure 1A, Tables S1 and S2.

### 2.2. Differential Gene Expression Analysis

To conduct the analysis of differential gene expression, initial normalization of gene expression values was performed using the DESeq2 R package (version 1.38.2) [9], where counts per million reads mapped (CPM) were utilized. For the Chikungunya dataset, CPM values were directly provided by the authors. In the case of the Dengue dataset, values were computed subsequent to mapping the sequences to the human genome (GRCh38.p13 release 104) employing STAR software (version 2.7.10a) [10]. Following alignment, the reads were summarized into count matrices utilizing the R package Rsubread (version 2.0.1) [11]. Differential expression analysis was then conducted using the R package DESeq2 (version 1.38.2) to identify genes exhibiting differential expression. The analysis is summarized in Figure 1B.

### 2.3. Cell Abundance, CDR3 Diversity, and Gene Set Enrichment Analysis

The Immune Cell Abundance Identifier (ImmuCellAI) tool [12] was employed to infer the abundance of immune system cells from counts per million (CPM) values. Sequences of the heavy-chain complementary-determining region 3 (CDR3), representing the entire functional Ig heavy-chain (IgH) repertoire, were acquired for T-cell receptors (TCRs) and B-cell receptors (BCRs) using MiXCR software (version 3.0.13) [13]. TCRs encompass TCRα (TRA) and TCRβ (TRB), while BCRs include heavy (IGH), kappa (IGK), and lambda (IGL) chains.

Diversity analysis of CDR3 sequences for each chain was conducted utilizing the immunarch R package (version 0.9.0) [14], with Chao1 as the diversity estimator. Pearson correlation coefficients were computed to evaluate the association between variance stabilizing transformation count data (VST) and CDR3 diversity values, using by the Hmisc R package (version 4.5.0) [15]. These coefficients were subsequently utilized in Gene Set Enrichment Analysis (GSEA) using the fgsea R package (version 1.24.0) [16], with gene modules sourced from the Molecular Signatures Database [17]. The normalized enrichment score (NES) from GSEA was calculated by normalizing the enrichment score (ES) for each gene set to account for differences in the gene set size. This normalization allows for comparisons across gene sets of different sizes. Specifically, NES was determined by dividing the actual ES by the mean of the ESs obtained from all permutations of the dataset, as described by Subramanian et al. (2005) [18].

## 3. Results

### 3.1. CDR3 Diversity and Cell Abundance

During the acute phase of CHIKV infection, individuals showed a reduced diversity of BCR and TCR CDR3 compared to healthy individuals (Figure 1C and Figure S1). In contrast, individuals infected with DENV exhibited a decreased diversity of TCRs only (Figure 1C and Figure S2). Similarly, the abundance of T cells in the blood was lower in both CHIKV- and DENV-infected patients, while the abundance of B cells was lower only in CHIKV-infected patients (Figure 1D and Figure S3).

### 3.2. Gene Set Enrichment Analysis

**Figure 2:**
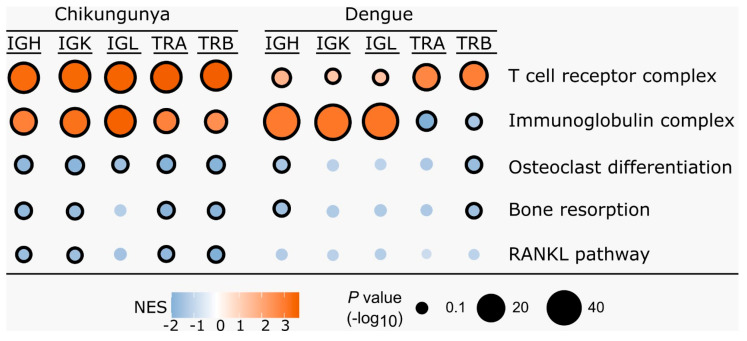
Gene set enrichment analysis of the correlation between gene expression and CDR3 diversity. The dot plot shows the normalized enrichment score (NES) for each gene set, which reflects the correlation coefficients between gene expression and CDR3 diversity. T-cell receptor complex and immunoglobulin complex are included as positive controls for TCR and BCR diversity, respectively. Circle size reflects the number of genes that contribute to the enrichment. Circles with black borders indicate an adjusted *p*-value < 0.1.

Gene Set Enrichment Analysis (GSEA) identified gene sets that were correlated with CDR3 diversity. Some of these pathways were related to osteoclast differentiation and activation, negatively correlated with CDR3 diversity, and enriched primarily in the Chikungunya dataset, as shown in Figure 2.

### 3.3. Transcriptomic Changes in Peripheral Blood during CHIKV Infection

**Figure 3:**
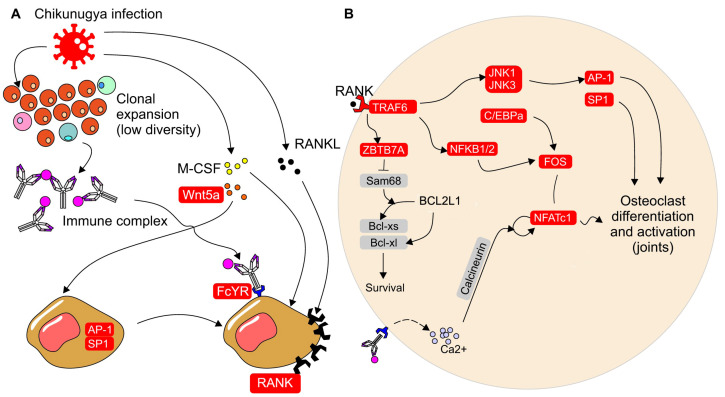
CHIKV infection increases the expression of genes associated to osteoclast differentiation. Proposed model of how CHIKV infection increases the expression of genes associated with osteoclast differentiation and activation. (**A**) WNT5A expression is elevated during CHIKV infection, leading to an increased RANK expression in osteoclast precursors through AP-1 and SP1 signaling. CHIKV infection also reduces BCR and TCR CDR3 diversity and may result in the formation of immunocomplexes that bind to FCyR. Osteoclast precursors that overexpress RANK and are stimulated by FCyR, RANK, and M-CSF receptors undergo differentiation and activation. (**B**) RANK signaling in osteoclast precursors ultimately leads to NFATC1 expression and osteoclastogenesis. Additionally, ZBTB7A expression promotes cell survival by favoring the alternative splicing of Bcl-xl, an antiapoptotic protein. Signaling through FCyR can increase intracellular calcium levels, which enhances NFATC1 expression. Genes upregulated during CHIKV infection are highlighted in red.

Genes associated with osteoclast differentiation and activation were upregulated in CHIKV-infected individuals compared to non-infected individuals, as shown in Figure 3A,B. The expression of these genes is consistently increased in CHIKV-infected individuals, but not in Dengue-infected individuals (see Table 1).

## 4. Discussion

The inclusion of the Dengue dataset demonstrates that the upregulation of osteoclastogenic genes correlated with immune repertoire diversity is a phenomenon specific to Chikungunya infection; another viral infection—in this case, Dengue—does not induce the same response as Chikungunya.

During the acute phase of CHIKV infection, patients show lower CDR3 diversity (Figure 1C and Figure S1). This is a common observation in infections due to the expansion of lymphocytes after antigen contact [19]. Lymphopenia, a characteristic of the acute phase of CHIKF [20], may explain the lower diversity observed in CHIKV-infected patients. Lower frequencies of B and T cells (Figure 1D and Figure S3) were also observed in CHIKV-infected patients, which could lead to a lower number of BCR and TCR reads and underestimate the diversity [21].

GSEA analysis revealed a negative correlation between CDR3 diversity and the expression of genes associated with osteoclast differentiation and activation pathways in CHIKV-infected individuals. This suggests that these genes are more highly expressed during the acute phase of infection (Figure 2).

Individuals infected with CHIKV show an upregulation of the receptor activator of nuclear factor-κB (RANK), macrophage-colony stimulating factor (M-CSF), and FcyR, which are involved in the induction of osteoclast differentiation and activation [22] (Figure 3 and Table 1). Additionally, WNT5A expression is upregulated, and has been demonstrated to stimulate the overexpression of RANK in osteoclast precursor cells through JNK, SP-1, and AP-1 signaling [23]. The activation of RANK and its ligand RANKL is crucial for osteoclast differentiation, as it leads to the expression of the Nuclear Factor of Activated T-Cells 1 (NFATC1) in precursor cells [24]. Upon RANK stimulation, intracellular signaling is initiated, involving the recruitment of TRAF6 and the activation of the MAP kinases (MAPKs) and NF-κB pathways. The MAPK pathway induces the expression of Ap-1, while the NF-κB pathway induces the expression of FOS [25]. Signaling through RANK also promotes the expression of C/EBPα (CCAAT/enhancer binding protein α) in osteoclast precursors [26]. Collectively, Ap-1, FOS, and C/EBPα facilitate the expression of NFATc1, which in turn stimulates the expression of genes responsible for osteoclast differentiation [22]. The activation of FCyR can also increase intracellular calcium concentration, further promoting NFATc1 expression [22]. In CHIKV-infected individuals, not only are the expression levels of RANK and NFATC1 upregulated, but also the other genes involved in these pathways (Figure 3 and Table 1).

The literature on RA combined with the results produced by our own analyses leads to the hypothesis that the chronicity of CHIKV infection may be linked to a higher number of circulating osteoclast precursors during the acute phase of infection, which are more resistant to apoptosis. These are observed in RA [27,28] and could be driven by Ig and T cells through a persistent ligand [29]. These resilient osteoclasts can migrate to the joints and remain activated, leading to chronic joint inflammation. RANK signaling induces the expression of ZBTB7A in osteoclast precursors, which in turn promotes the overexpression of NFATC1 and favors the alternative splicing of the BCL2L1 gene into Bcl-xl, an anti-apoptotic protein. Xu et al. (2022) demonstrated that ZBTB7A overexpression leads to the degradation of Sam68, which favors alternative Bcl-xl splicing and longer osteoclast survival. Moreover, ZBTB7A expression is upregulated by cytokines in inflammatory conditions. ZBTB7A expression is also increased in CHIKV-infected individuals during the acute phase of infection (Figure 3B and Table 1) [30].

Because there is little to no bone erosion associated with CHIKF [31], the mechanism that generates chronic joint pain must be distinct from RA. Osteoclasts activity is also seen in viral infections not associated with erosive arthritic symptoms [32,33]. Additionally, osteoclasts perform activities not related to bone erosion [34]. Lower CDR3 diversity and higher expression of genes involved in osteoclastogenesis may be linked to the persistent presence of antigens. Osteoclasts are activated by FcγR through IgG antibodies bound to their antigens in the form of immunocomplexes, which promotes their differentiation and activation [35,36]. In acute CHIKF, lymphocytes may undergo clonal expansion after contact with CHIKV antigens, followed by antibody production and larger pools of monoclonal T cells. Antibodies activate FcγR signaling in osteoclast precursors, enhancing osteoclastogenesis. Due to the nature of CHIKV infecting several cell types sequentially, including macrophages and synovial cells, the inflammatory response can become sustained. This is also furthered by the T-cell cytokine-mediated recruitment of host cells to synovial tissues, sustaining ligands and promoting sustained B-cell antibody production at high circulating levels [37]. Ultimately, these factors can contribute to joint pain and the development of the chronic phase of CHIKF.

The findings presented in this study stem from analyzing the transcriptome of peripheral blood mononuclear cells (PMBCs) from patients infected with CHIKV. Consequently, discriminating the specific cell types responsible for the observed changes in gene expression poses a challenge, particularly for genes expressed across various cell types. The reliance on peripheral blood samples may not fully capture the molecular changes occurring in joint tissues, where the manifestations of CHIKV chronicity are most pronounced. To delve into this further, investigating the presence and prevalence of osteoclast precursors, along with their expression patterns during CHIKF, requires both in vivo and in vitro investigations, utilizing tissue samples or in vitro models. These investigations would include techniques such as flow cytometry, cell sorting, single-cell sequencing, and assays focused on osteoclastogenesis. Additionally, the limited information available on the patients included in these datasets restricts our ability to control for individual variability and fully contextualize the findings.

If the hypothesis suggesting osteoclast involvement in CHIKF proves correct, existing interventions utilized for other conditions might offer potential benefits for both the acute and chronic phases of CHIKF. One such intervention is the monoclonal antibody denosumab. It works by binding to RANKL, thereby preventing its interaction with RANK, which halts the process of osteoclast differentiation and activation [38]. Furthermore, if immune complex formation plays a role in osteoclast activation during CHIKF, a possible treatment avenue involves reducing FcγR signaling by promoting IgG sialylation [9]. Lastly, inducing osteoclast apoptosis, a strategy already successful in treating arthritis, could also serve as a viable option for managing chronic joint pain associated with CHIKF [39].

## 5. Conclusions

In summary, the analysis conducted in this study reveals a significant reduction in BCR and TCR CDR3 diversity during the acute phase of CHIKV infection, which correlates with an upregulation of osteoclastogenic genes in peripheral blood mononuclear cells. This suggests that the immune dysregulation induced by CHIKV may contribute to the persistence of joint pain and the development of chronic arthralgia in some patients. Notably, the enhanced expression of genes involved in the RANK/RANKL signaling pathway indicates a potential mechanism through which CHIKV infection may promote osteoclast survival and activity, leading to prolonged inflammatory responses in joint tissues. These insights help us to further explore the underlying mechanisms of CHIKF chronicity and highlight potential therapeutic targets, such as the inhibition of osteoclastogenesis, to mitigate long-term joint damage and pain in affected individuals.

## Figures and Tables

**Table 1:** Differentially expressed genes in CHIKV- and DENV-infected samples.

Gene	CHIKV log2FoldChange	CHIKV padj	DENV log2FoldChange	DENV padj
CEBPA	0.72	0.000033	−0.12	0.5844875
FCER1G	2.08	0.000000	1.91	0.0000000
FOS	1.90	0.000000	0.08	0.8726659
JUN	2.99	0.000000	−0.43	0.0368901
MAPK10	0.68	0.003251	1.96	0.0378769
MAPK8	0.31	0.000891	−0.52	0.0005112
NFATC1	0.81	0.000330	−0.01	0.9390283
NFKB1	0.52	0.008601	0.14	0.4168015
NFKB2	2.29	0.000000	0.47	0.0129732
RELA	1.34	0.000000	0.37	0.0119984
SP1	0.37	0.004499	0.59	0.0000375
TNFRSF11A	0.94	0.004819	−0.05	0.9545348
TRAF6	1.48	0.000000	0.39	0.0382718
WNT5A	3.01	0.000518	-	-
ZBTB7A	1.55	0.000000	0.11	0.3841900

## Data Availability

The datasets used here can be downloaded from the following links: https://www.ncbi.nlm.nih.gov/bioproject/PRJNA507472 (accessed on 13 May 2020) and https://www.ncbi.nlm.nih.gov/pmc/articles/PMC8578928/ (accessed on 19 October 2020).

## Supplementary Materials

The following supporting information can be downloaded at: https://www.mdpi.com/article/10.3390/idr16050073/s1, Figure S1: CDR3 diversity is lower in CHIKV-infected samples; Figure S2: T-cell CDR3 diversity is lower in DENV-infected samples; Figure S3: Changes in the cellular abundances of T and B cells in Chikungunya and Dengue virus infections; Table S1: Meta information for CHIKF patients and controls; Table S2: Sample status and gender for Dengue virus infection.
