# Supplementary material for: Chikungunya-Driven Gene Expression Linked to Osteoclast Survival and Chronic Arthralgia
Source: Infect Dis Rep. 2024 Sep 20;16(5):914–22. doi: 10.3390/idr16050073 (PMC11417755; doi:10.3390/idr16050073)
Supplement: Supplementary file 1 [file idr-16-00073-s001.zip › supplementary/supplementary.docx]

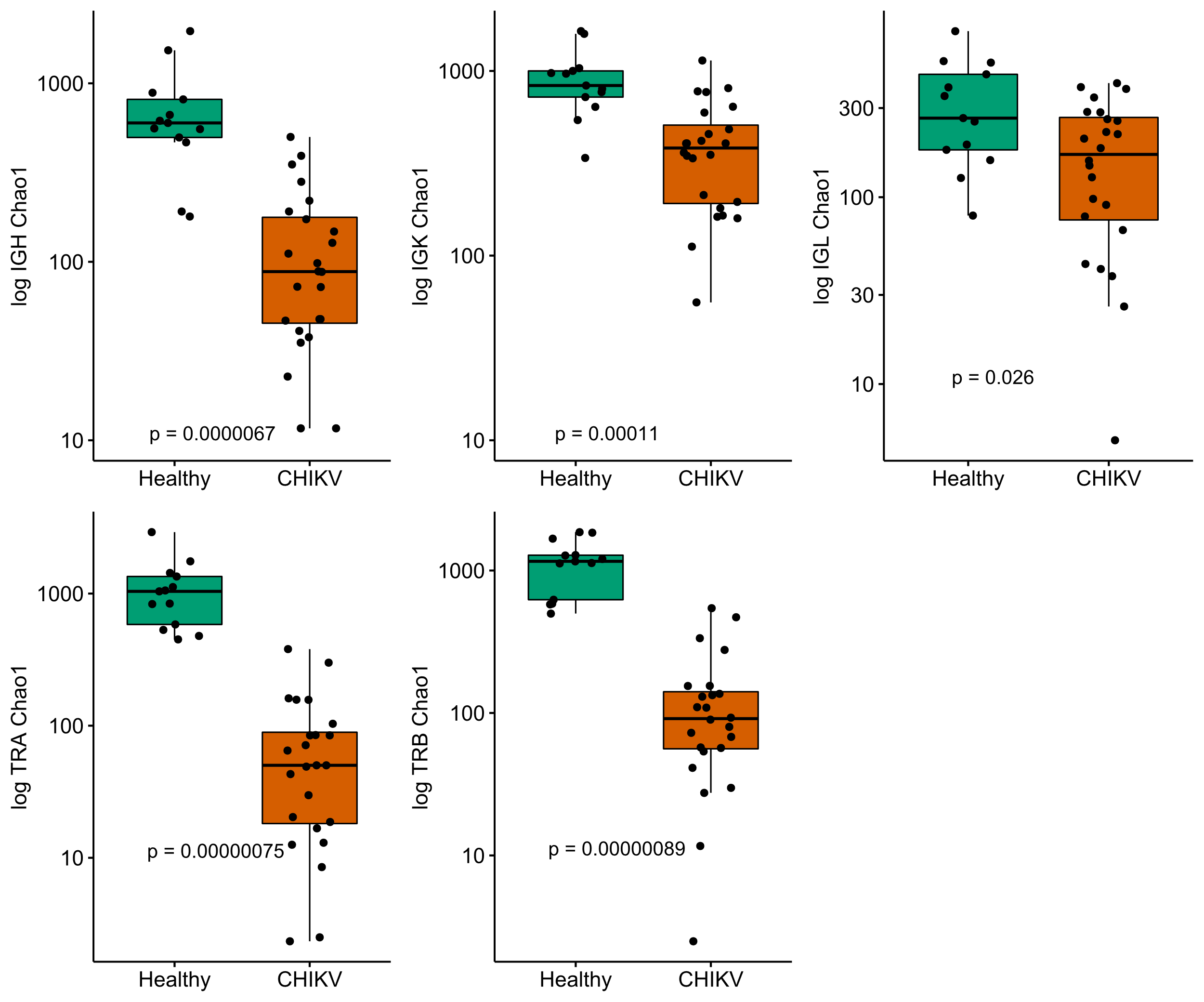

**Figure S1.** CDR3 diversity is lower in CHIKV-infected samples. Estimated Chao1 diversity for B cell (IGH, IGK, and IGL chains) and T cell receptors (TRA and TRB chains) of healthy (green) and infected with Chikungunya virus (orange) individuals. Wilcoxon-Mann-Whitney test *p*-values are shown for each comparison.


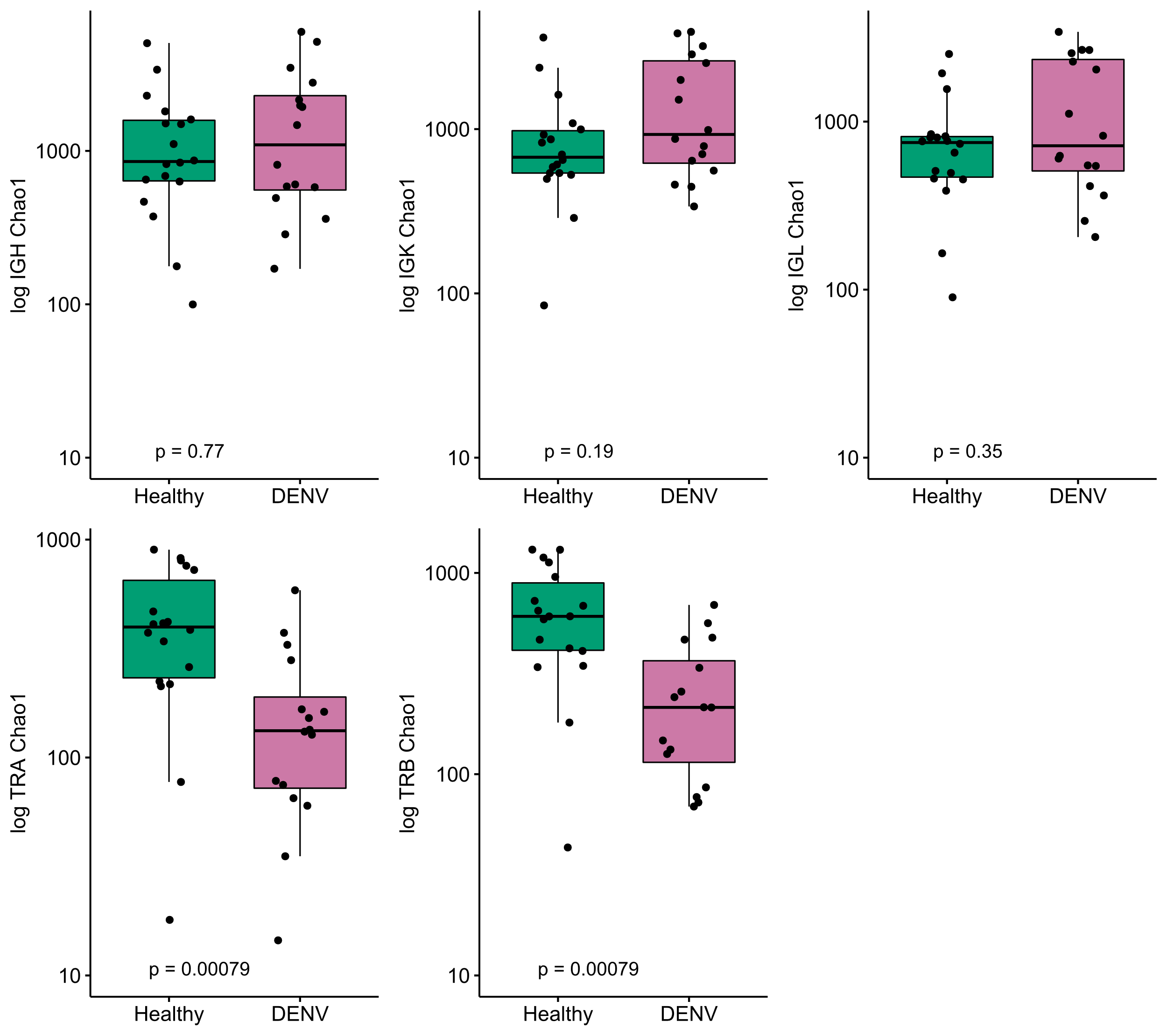


**Figure S2.** T cell CDR3 diversity is lower in DENV-infected samples. Estimated Chao1 diversity for B cell (IGH, IGK, and IGL chains) and T cell receptors (TRA and TRB chains) of healthy (green) and infected with Chikungunya virus (orange) individuals. Wilcoxon-Mann-Whitney test p-values are shown for each comparison.

**
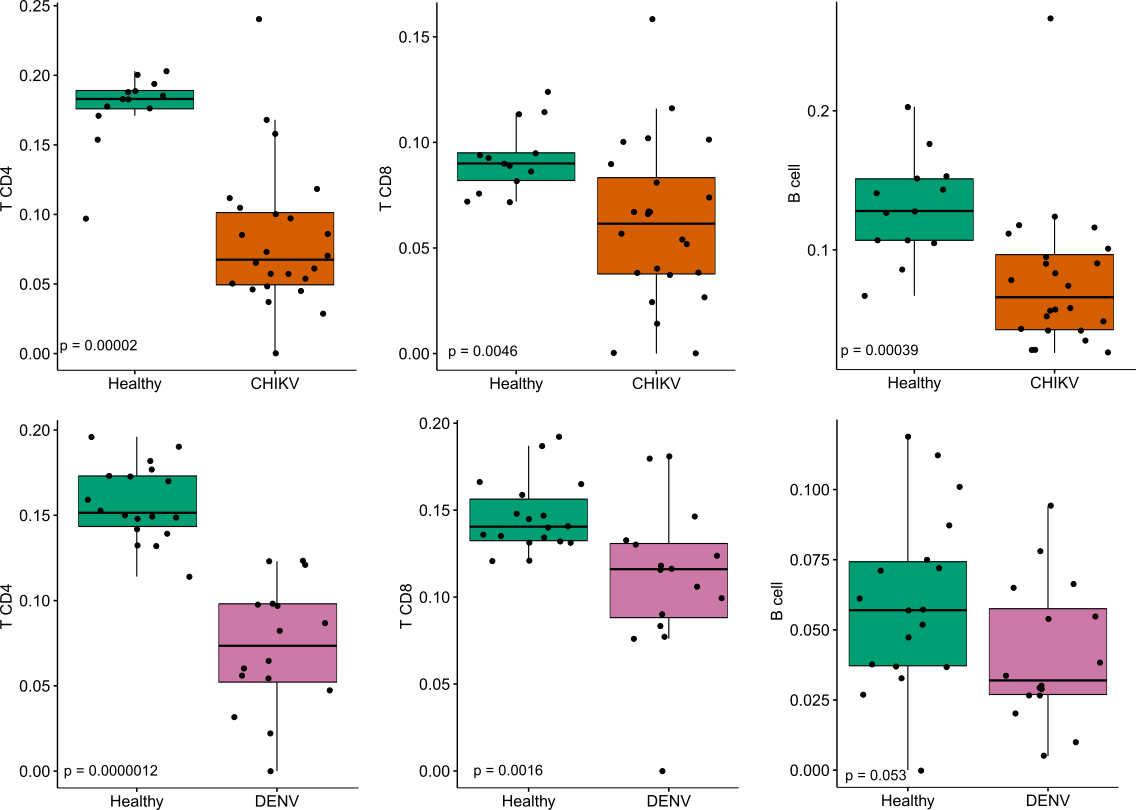
**

**Figure S3.** Changes in the cellular abundances of T and B cells in Chikungunya and Dengue virus infections. Estimation of cell proportions in samples from healthy individuals (green), Chikungunya-infected individuals (orange), and Dengue-infected individuals (pink). Wilcoxon-Mann-Whitney test *p*-values are shown for each comparison.
